# The Use of Geotechnical Methods to Determine the Deformation Parameters of the Ground in Terms of Operation and Safety of Mortar Use

**DOI:** 10.3390/ma14237237

**Published:** 2021-11-26

**Authors:** Grzegorz Bartnik, Kazimierz Józefiak, Małgorzata Superczyńska, Magdalena Czerwińska, Witold Krajewski, Józef Legieć, Tadeusz Kuśnierz, Mariusz Magier

**Affiliations:** 1 Institute of Roads and Bridges, Faculty of Civil Engineering, Warsaw University of Technology, 16 Armii Ludowej Ave., 00-637 Warsaw, Poland; G.Bartnik@il.pw.edu.pl (G.B.); k.jozefiak@il.pw.edu.pl (K.J.); M.Superczynska@il.pw.edu.pl (M.S.); 2 Military Institute of Armament Technology, Prym. S. Wyszyńskiego 7 Str., 05-220 Zielonka, Poland; czerwinskam@witu.mil.pl (M.C.); krajewskiw@witu.mil.pl (W.K.); legiecj@witu.mil.pl (J.L.); kusnierzt@witu.mil.pl (T.K.); 3 Institute of Mechanics and Printing, Faculty of Mechanical and Industrial Engineering, Warsaw University of Technology, Narbutta 85 Str., 02-524 Warsaw, Poland

**Keywords:** mortar, operational safety, organic soils, weak soils, deformation modulus, dynamic deformations

## Abstract

During firing from a mortar, an important issue is the parameters of compressibility of the ground on which the mortar is placed. This affects the operation of the mortar (including safety). During the qualification tests of the mortar, the influence of different types of terrains on its strength and work during shooting should be examined. Until now, in the Polish standardization documents there was no clear description of the ground parameters used for these kinds of tests. Analysis of the literature also did not allow to determine the dependence of the mortars displacement in the function of the type of ground and its geotechnical parameters. In view of the above, it has become important to draw up a research problem in the form of determining the types of soil with parameters, enabling the mortar tests to be carried out in conditions as close as possible to combat conditions. Therefore, the authors carried out the theoretical calculations and field tests with the use of geotechnical methods such as static and dynamic load tests to determine the parameters of the ground for mortar testing. Preliminary tests were conducted using the prescribed measurement methods and a comparative mortar firing test. Subsequently, an analysis of the results was carried out and the possibilities of using the tested methods of measuring the parameters of soil compressibility were determined.

## 1. Introduction

Infantry mortar is an fundamental weapon of modern warfare. Across the history of the weapon, it has enabled the infantry to have their own artillery support. The infantry mortar as developed after World War I has remained basically the same. The requirements of modern troops, including high mobility, significantly changed the weight of the mortar, but did not change its general combat characteristics. The fire task of mortars from the beginning of their existence has remained practically unchanged—firing from a high angle of the trajectory at the target in order to support the infantry. In addition, mortars play an important role in countering terrorist threats in the case of cover activities of operational bases and convoys in enemy areas of operation. Modern mortars (due to increased range, precision, and effectiveness) allow infantry units to effectively and precisely fight targets even in urbanized areas. Compared with other types of artillery equipment, they are characterized by the following characteristics:

- Shooting is carried out at the angles of the sight c ≥ 45°;
- The recoil force created during the shot is transmitted via the base plate directly to the ground. The towed mortar does not have any devices that would absorb (decrease) the impact of the recoil force pulse on the base plate;
- In the majority, mortars have a smoothbore tube (barrel). For this reason, the projectiles fired from mortar are fin-stabilized [1,2,3,4,5].

Since the mortars are moving with the maneuver element (infantry), they can see friendly forces and are often within close distance of the battlefield leadership. This allows them to shift fire as friendly troops advance and hit changing target priorities in real short time.

**Figure 1:**
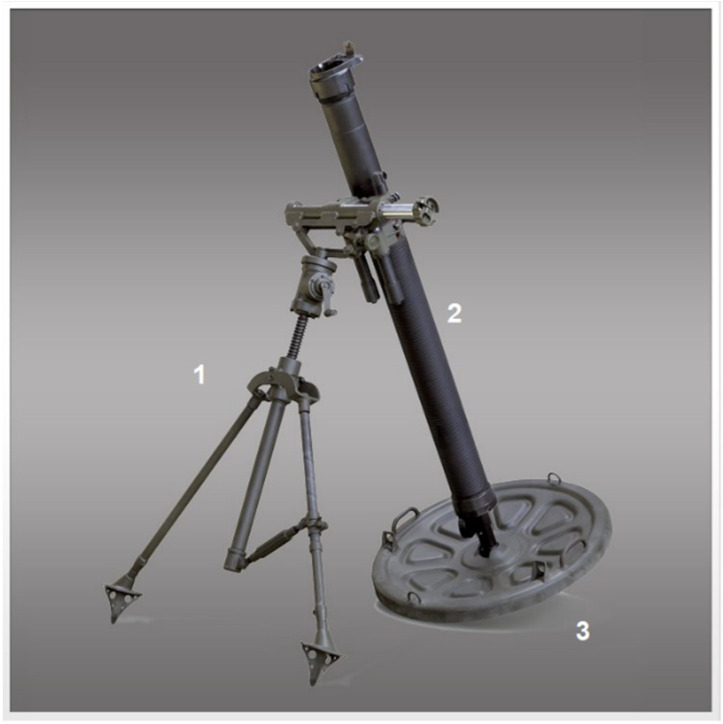
Mortar M98 (HSW S.A.), 1—bipod unit with elevation and direction mechanisms, 2—mortar tube, and 3—base plate.

A mortar with a typical design for this type of weapon used by Polish Land Forces is a 98 mm M98 (Figure 1) mortar manufactured by Huta Stalowa Wola S.A (Stalowa Wola, Poland) [6].

The mortar tube is used to give the required velocity and flight direction to the projectile. For the correct positioning of the tube, the bipod assembly is used. The base plate is used to support the mortar during the shot and transmit the recoil impulse to the ground.

The main problem with current infantry mortars is that they do not have a mechanism to reduce the recoil force or energy-absorbing recoil of the mortar assembly after firing a shot. It is not uncommon for a mortar to jump close to 1 m above the ground when placed on a non-absorbing surface. This presents a significant risk to mortar operators [7].

The issues of interaction between the mortar base plate and the ground are not widely described in world literature. The main emphasis in research is on the analysis of the stress and strength of the mortar itself (including the base plate). The paper [8] discusses studies of the effort in various elements of the mortar during shooting from gravel ground. Tests of this type allow to determine the safety of use (strength) of the mortar. The paper [9] presents a shotless low-cost method of analyzing the strength of a base plate. Such research is the basis for optimizing the design of the mortar. The paper [10] shows an example of optimizing the construction of a mortar’s base plate in terms of reducing its weight while maintaining the required strength and rigidity. The weight of the mortar is a very important parameter because it is supposed to be a light and mobile weapon.

Studies on mortar recoil have been presented for pyrotechnic mortars at work [11], but they show the results of the much lower loads than military mortars. For the issues of the relationships between the mortar and the substrate with different parameters, the authors did not find any studies. According to the authors, it is important to solve the research problem consisting in determining the types of soil that will be suitable for testing mortars in close combat conditions. It is particularly important from the point of view of conducting field tests of the new mortars intended for the army. Additionally, appropriately selected research methods and field tests will be provided to determine the influence of the essential factors on the results obtained and to determine a correlation between the results obtained by means of the presented research methods and shooting tests.

## 2. Dynamics of Mortar Movement during the Firing Process

**Figure 2:**
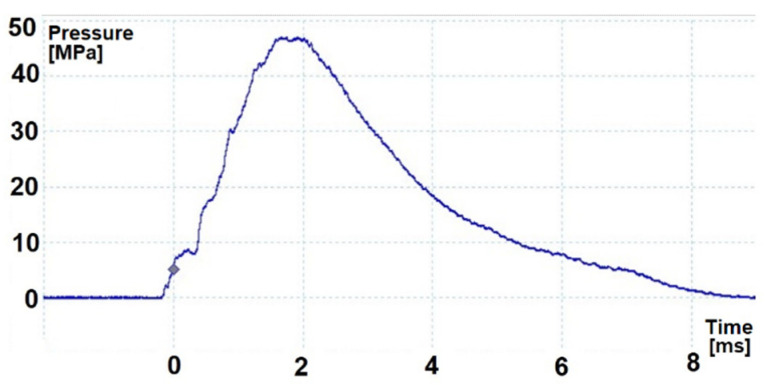
Example of pressure *p*(*t*) in the tube of a 60 mm mortar during firing.

**Figure 3:**
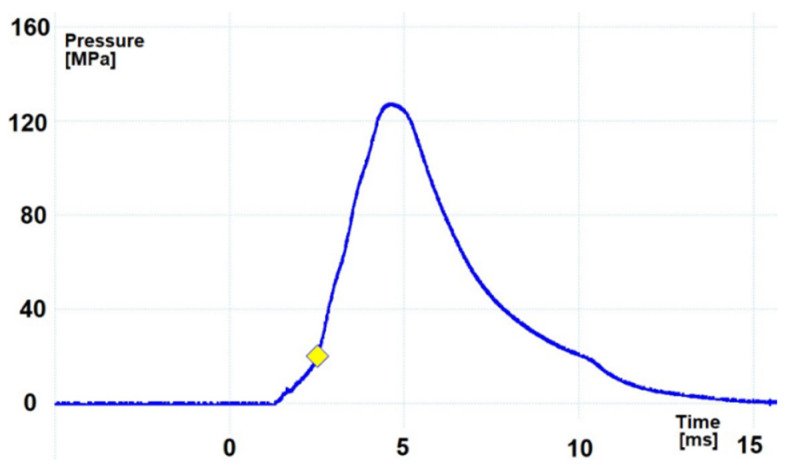
Example of pressure *p*(*t*) in the tube of a 98 mm mortar during firing.

In order to give the mortar projectile an initial velocity that would provide the required distance, appropriate propelling charges shall be used. These charges during the shot burn create a high pressure of gunpowder gases that affect both the projectile and the bottom of the barrel. Although the mass of the propelling charge is variable (depending on the required distance of the projectile), in this article the work of the mortar on the full propelling charge will be described, ensuring both the highest velocity of the projectile and the highest pressure of gunpowder gases inside the tube [12]. Figure 2 and Figure 3 show the pressure *p*(*t*) diagrams inside the 60 mm and 98 mm mortar barrel tube during firing (which were obtained during verification tests of mortars conducted in the Military Institute of Armament Technology).

In the case of a 60 mm mortar, the maximum pressure shown in Figure 2 is about 47 MPa which, after taking into account the surface of the bottom of the barrel of ≈0.002827 m^2^, generates a maximum force acting on the mortar ≈134.3 kN. For a 98 mm mortar, these are about 127 MPa, ≈0.007543 m^2^, and ≈958 kN, respectively. Because the pressure in the tube and the associated forces acting on the mortar are quickly changing, most often the total impulse of momentum force transmitted to the gun during the shot is used to describe the recoil phenomenon. Table 1 presents various parameters related to the phenomenon of recoil in mortars operated by the Polish Armed Forces when firing on full charges. An impulse of the recoil force is understood as the momentum transmitted to the mortar elements participating in the recoil. The theoretical recoil velocity is the value that the mortar elements would reach during recoil if they were not affected by braking forces.

**Figure 4:**
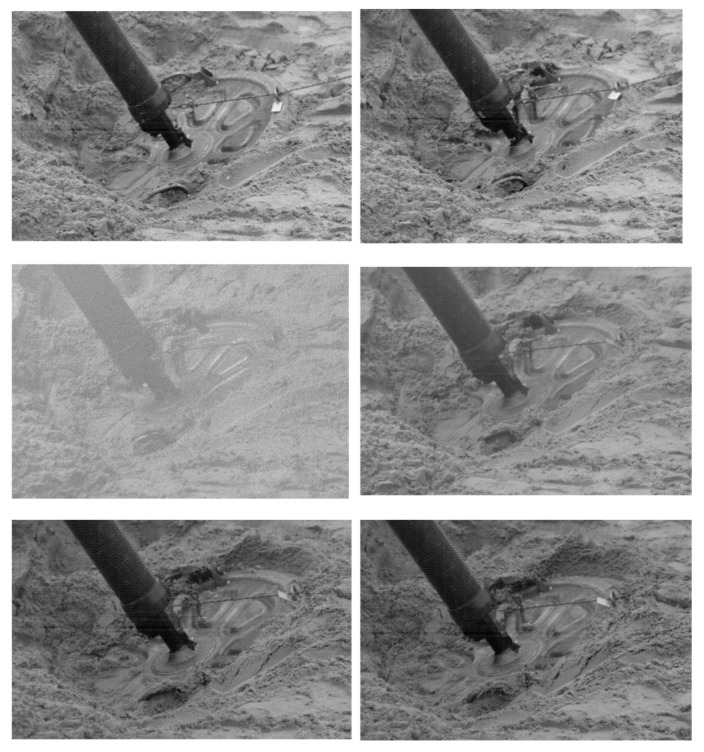
Mortar’s recoil process on sandy soil.

To brake the barrel of the mortar and ensure its stability during the shot, a base plate is used, which transmits the recoil force to the ground. Due to the much larger diameter of the base plate in relation to the diameter of the barrel, the pressures with which the base plate can affect the ground are much smaller. Nevertheless, for a 60 mm mortar, the maximum pressure with which the base plate can affect the ground is 1.57 MPa. In the case of a 98 mm mortar, it is 1.9 MPa. In most cases, the shooting is carried out by the mortar adapted to the deformable substrate and it will delve into the ground together with the base plate during the shot. Figure 4 shows a sequence of frames from a movie, recorded with a high speed camera during a mortar shot, showing the sinking of the base plate into the sandy soil. Figure 4 shows the frames recorded every 10 ms.

For a terrain susceptible to deformation during firing, the resultant force, which is the sum of the recoil force and the reaction force of the terrain on the base plate, creates the mortar’s velocity in the opposite direction to the projectile velocity, which causes the mortar to plunge into the ground. After the projectile leaves the tube and the pressure of the gunpowder gases in the barrel decreases to the level of the atmospheric pressure, the force of inertia of the mortar causes its further recess into the ground [13,14,15,16]. At this stage, the only significant force acting on the mortar is the reaction force of the ground, which inhibits its recoil speed. After stopping the mortar, as a result of the ground response, the mortar may move in the opposite direction, which is called “jumping”.

The quality requirements for mortars stipulate the possibility of firing from them after placing on grounds with a wide range of properties. Any proper interaction of the mortar and the terrain is largely dependent on the latter’s deformation modulus [17,18]. For rigid substrates the “jumping” of the mortar and its displacement or sliding off the base plate onto the ground may occur. With a terrain very responsive to deformation, the mortar together with the base plate can sink into a significant (relative to the mortar) depth or slip away. A large displacement of the mortar when delving into liable ground or during its “jumping” on a non-liable and elastic ground significantly prolongs the fulfillment of the fire mission because before each shot it is required to re-set the mortar. A significant influence on the results of mortar shooting will also have its stability during the shot (especially during the movement of the projectile inside the gun tube). Even a slight deviation of the mortar barrel (by 1°) in the direction will cause a deviation of the point of hit of the projectile (at a firing distance of 2000 m)—by over 32 m, and with a shooting distance of 5000 m—by over 80 m [19]. The impact of the mortar tube’s deflection in the vertical plane is several times smaller. Therefore, artillery practice often requires (in the case of placing the mortar on susceptible ground) firing a first shot (called the “setting shot”) in order to place the mortar in the ground.

Another important aspect resulting from the interaction between the mortar and the ground is the safety and reliability. Mortar firing on a low-susceptible ground significantly increases the strain in the main mortar’s parts. This applies in particular to the base plate and the connection between the base plate and the barrel. Therefore, the development of a base plate resistant to multiple impulse loads, and at the same time ensuring stability of the mortar when placed on various types of terrains, requires determining the relationship between the base plate and the ground during the shot. When firing from a very susceptible ground, a large displacement of the mortar may lead to damage of the barrel, the barrel support, or both, or, in extreme cases, because of the slipping of the base plate, cause the mortar to overturn. Thus, heavy (200 kg or more) mortars of larger calibers, may pose a threat to the safety of the crew.

## 3. The Issue of Interaction between Mortar and the Ground from the Standardization Point of View

The issue of interaction between mortar and the ground significantly affects the problem of safety and reliability of the mortar use. It is regulated mainly by the Defense Standards, which is a series of documents supervised by the Polish Military Center for Quality Standardization and Codification for the normalization of issues related to weapons equipment. The standard NO-10-A216:2012 [20] contains a set of requirements and tests that must be fulfilled by mortars introduced into the Armed Forces.

Because operational safety is an overriding requirement for armament equipment, the standard NO-10-A216:2012 [20] clearly requires the safety and correct use of mortars placed on various types of ground. The mortar test procedure requires conducting research shooting on “soft” and “hard” soils. The following types of soil are determined:

1. Hard soils (calcareous (2500 kg/m^3^), stony (2900–3200 kg/m^3^), compacted clay (1770 kg/m^3^), and frozen soils (620–2000 kg/m^3^));
2. Medium soils (sandy (1620 kg/m^3^), clayey (1500–1800 kg/m^3^), and turf soils (1500 kg/m^3^));
3. Soft soils (muddy (1840 kg/m^3^), “arable” soils (1300 kg/m^3^), and peat and “plump sand” (1440 kg/m^3^)).

Preliminary analysis of the issue showed that the greatest complication occurs in the case of soft soils. Apart from the inconsistency in nomenclature, the above-mentioned names of soft soil types do not appear in the geotechnical standards [21,22]. The soft soils in the engineering practice should be treated as low, with insufficient bearing capacity. “Weak soil” may be defined as a soil layer that does not fulfill the requirements concerning compressibility, stability, or suitability for use for a particular object or element of structure. In engineering practice, the parameterization of weak soils is practically limited to determining their depth of deposition in the active zone and usually they are not described by the values of geotechnical parameters. They are reinforced in a way that depends on the depth of deposition, the type of soil, and designed construction. Another aspect that makes geotechnical investigation of the ground difficult is the limitations resulting from the technical capabilities of the research equipment, which is not adapted to determine the parameters of weak soils. Moreover, there are no appropriate standards to interpret the obtained results of these types of soil tests. In addition, when choosing a test method, the conditions of the proving ground should be taken into account as a place where test shooting will take place and where it is necessary to prepare and examine the ground for its use as the shooting position. Therefore, the ground should:

- Allow easy preparation without specialist geotechnical (geological) knowledge and heavy equipment;
- Provide repeatability of obtained ground conditions;
- Be recognized by data that does not require the use of complicated or time-consuming methods and can be determined under field conditions;
- Allow to prepare the ground in a short time;
- Be easily obtainable.

## 4. Soft Soil

### 4.1. Selection of a Model Substrate

The standard [20] introduces the following types of soft soils: muddy, arable soils, peat, and plump sand. Taking into account the potential difficulties with the use of cohesive soils as a weak layer during their preparation, formation, and compaction resulting from the need to moisten them (to achieve at least a plastic state), the modeling of “muddy soil” or “arable soil” was omitted. It was decided to use “peaty soil” (resigning from modeling “plump sand” due to the possibility of obtaining a much weaker soil with a wider range of “weaknesses”), which is widely available for gardening use. Peat is a demanding research material; however, it maintains constant parameters in various weather conditions and is widely available. The peat used in this study can be described as dry and fibrous (no decomposed organic parts) with clearly recognizable plant remains with no solids.

Initially, it was assumed that the substrate model would be characterized by parameters describing deformability. The bearing capacity of soils increases with compaction, then it is necessary to control the amount of peat to obtain the required values of deformation parameters. However, in engineering practice for peats there is no test procedure to determine the values of the state of compaction parameters, i.e., relative density (density index) *I_D_* or relative compaction (degree of compaction) *I_S_*.

The density index *I_D_* determines the condition of the cohesionless soils, which peat is not. In accordance with the standard EN 13286-2:2010 [23], there is no test procedure to determine the value of the maximum dry density of the peat ρ_ds_ which is taken into account in the calculation of *I_S_*. Determining the value of the ρ_d_ for peat is quite difficult due to the need to take a sample with an undisturbed structure. It is also not possible to use indirect methods for determining compaction parameters such as dynamic probing.

In the authors’ opinion, it is possible to determine the bulk density of the ground model and control this parameter while preparing the shooting post. It should be noted that the value of the density depends on the moisture, which in the case of peat can be within a wide range. The parameter that better describes the state of compaction is the dry density. This method requires the determination of the moisture value, which in field conditions can be checked using, e.g., the electric method (presupposes determination of soil resistance, conductivity, inductance, as well as capacity) or the tensometric method (based on the difference in the voltage of water between the phase boundaries).

### 4.2. Research Methods

The methods of assessing the deformability of the ground in situ include: the static plate load test and dynamic load plate test. These methods allow for direct measurement of the soil response to an applied load described by the following parameters: the deformation modulus *E* and the dynamic modulus of deformation *E_vd_*.

The static plate load test is commonly used in road construction in the form of loads generated by the VSS apparatus (Verein Schweizer Strassenfachmänner, Zürich, Switzerland). The deformation modulus in the VSS test is defined as the ratio of the unit load increment to the deformation increment of the tested layer in a fixed range of unit loads [24]. Double static loading of the test surface allows to calculate the primary E_1_ and secondary E_2_ deformation modules (initial and reload modulus, gained from static plate load test). In the case of assessment of the stiffness of the thick ground layers, large-scale load tests are used [25].

The dynamic load plate test by lightweight deflectometer was developed initially in Germany [26,27,28,29,30] as a device for in situ testing of stiffness and compaction of the ground and road construction layers, as well as an alternative to static testing. The main advantage of this method is mobility and short test time. In the dynamic load plate test, the amplitude of plate displacement caused by a single impact pulse is measured.

### 4.3. Test Stand

**Figure 5:**
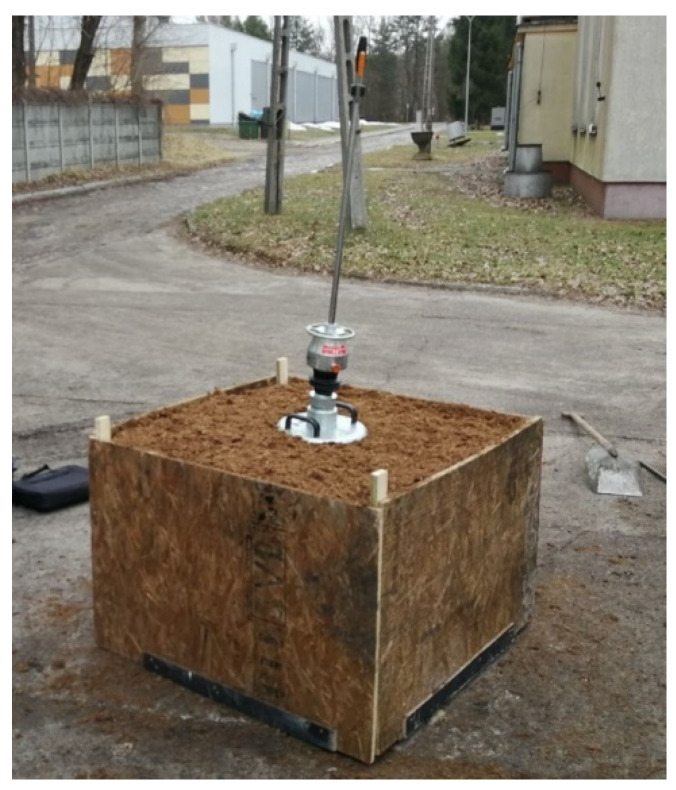
Test stand.

To assess the possibility of using peat as a model ground of the shooting station, a preliminary research program was planned and carried out. The program includes verification of the use of peat in the test stand and the validity of the selected research methods. For this purpose, a test stand was made in the form of a wooden box with a volume of 0.7 m^3^ in which previously weighed peat was placed. About 197.4 kg of peat was placed in the box, which allows to determine the bulk density at 282 kg/m^3^ (Figure 5).

### 4.4. Static Load Method

The static plate load tests were carried out using a standard rigid plate (diameter 30 cm) included in the VSS apparatus. In practice, due to the lack of space, the use of dial sensors and the rack attached to the apparatus for settling measurements has been omitted.

The load of ~215 kg consisted of a stack of 7 steel plates (20 ÷ 30 kg each) and a VSS plate. Applying the entire load was divided into 7 stages.

The measurement of displacement was carried out using the geodetic method at four points on the surface of the rigid plate. This allowed to eliminate possible stack tilts, and the average value of settlements measured at individual points was taken as a result.

The load consisted of two phases: the primary loading and the subsequent unloading. Each phase was carried out in stages, and the final load value was selected to obtain pressures similar to those generated by humans (approx. 30 kPa). It was assumed that in field conditions, human-induced subsidence could be used as an initial rating of the ground quality of the shooting position.

### 4.5. Dynamic Load Method

**Figure 6:**
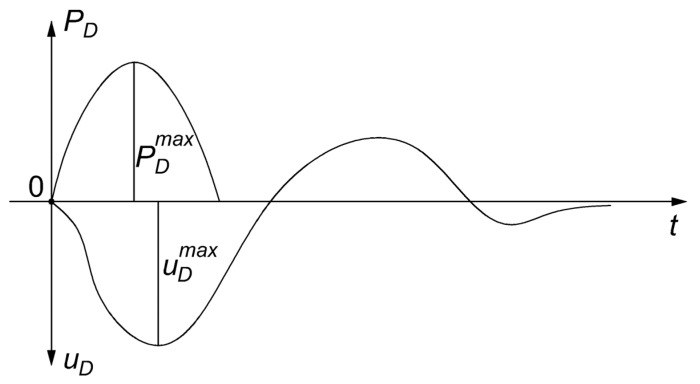
Variability in time t of loading force *P_D_* and displacement *u_D_* in a dynamic test [30].

The dynamic load plate test consists of measuring the deformation of the ground surface, caused by a short-term force impulse. The impulse load is generated by a freely falling mass and transmitted through the pressure plate to the ground. This method is treated as a short-term quasistical load of the plate on the ground. The free fall of the weight, falling along the guide rod on the spring-damper element, generates an impact-like load *P_D_* on the load plate (Figure 6). The pulse is transmitted through the load plate to the surface of the ground, generating a contact stress *σ_D_* = 100 kPa and causing the substrate settlement equal to the displacement *u_D_* of the rigid load plate [29].

**Figure 7:**
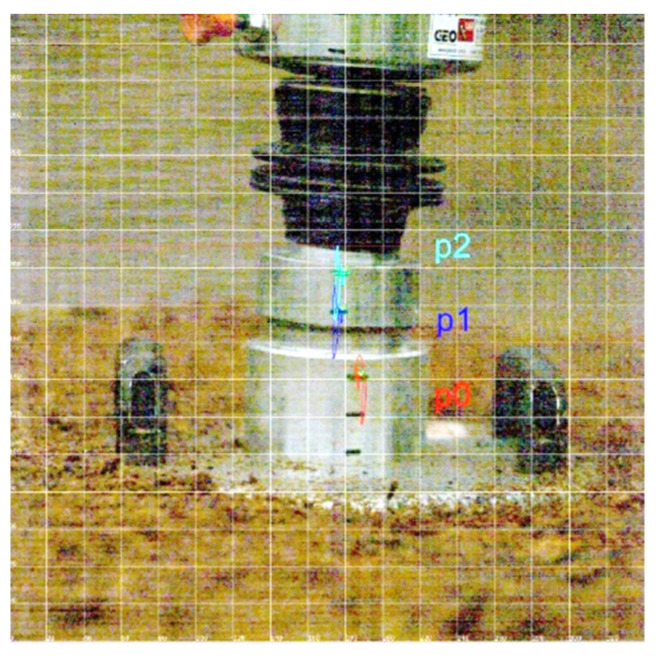
Displacement of reference points during impact.

In the research schedule, it was assumed that the determination of the dynamic modulus of deformation *E_vd_* will be carried out with a lightweight deflectometer ZORN ZFG 2000 (Zorn Instruments GmbH & Co. KG, Hansestadt Stendal, Germany). During the tests, it turned out that the magnitude of the generated acceleration exceeds the measuring capacity of the accelerometer amounting to ±100 g, which corresponds to a displacement of 0.2–30 mm ± 0.02 mm. Therefore, a Photron high-speed camera was used to determine the course of displacement during the dynamic test. The image was recorded at a frequency of 6000 frames per second, but the analysis of the dynamics of this phenomenon indicates that 2000 frames per second is sufficient. The recordings from the camera were subjected to imaging analysis in order to determine the changes in the position of the measuring points during the test. During one study, three drops were performed. The analysis took into account three reference points (p0; p1; p2) marked on the device used (Figure 7).

### 4.6. Results

Starting from the analysis of settlement of the soil as isotropic and homogeneous elastic half-space, the value of the substrate deformation modulus *E* from static plate load tests and the dynamic modulus of deformation *E_vd_* from dynamic plate load tests can be determined from the Equation [31]:

(1) $$E,\;E_{vd}=\left(1-\nu^{2}\right)\omega \frac{D\;\Delta p}{\Delta s}$$

where: *ν*—Poisson’s ratio, *ω*—the shape factor, which for a circular rigid plate takes the value *ω* = 0.79, *D*—diameter of the loading plate (S355J0 steel, Young’s Modulus is 190 to 210 GPa) (m), Δ*p*—the increase in load (Pa), and Δs—the increase in settlement for given increase in load (m).

For a dynamic load plate test Δ*p* = 0.1 MPa, Δ*s* is the maximum displacement of the loading plate.

**Figure 8:**
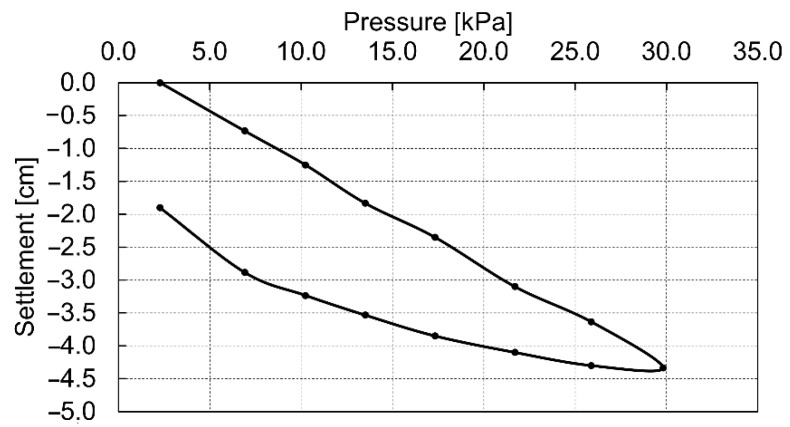
Pressure-settlement relationship for static load test.

The results of the static tests, calculated using Equation (1), are shown in Table 2. Figure 8 presents the dependence of the soil settlement on the used loads. The shape of this graph suggests the linear soil response to the applied loads. In this case, in the calculation of the modules, the value of the Poisson’s ratio for peat ν=0.2, and the plate diameter D=30 cm were assumed.

The values of the primary static deformation modulus for each stage are listed in Table 3.

**Figure 9:**
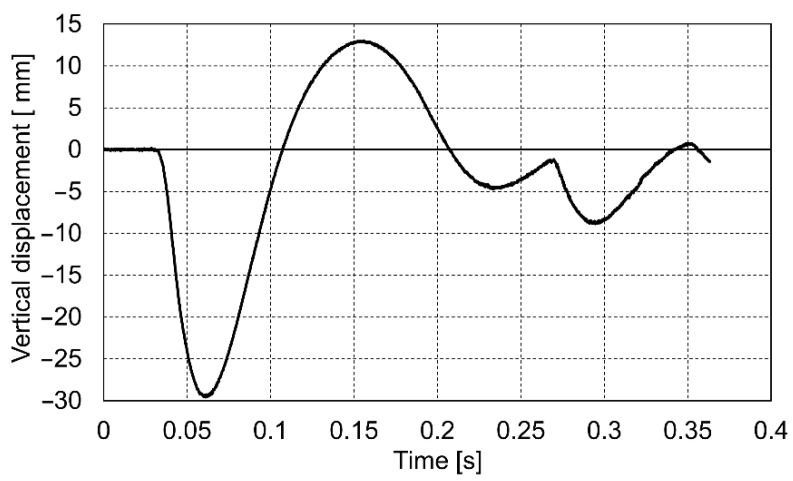
Vertical displacement of the p0 point during drop No. 1.

**Figure 10:**
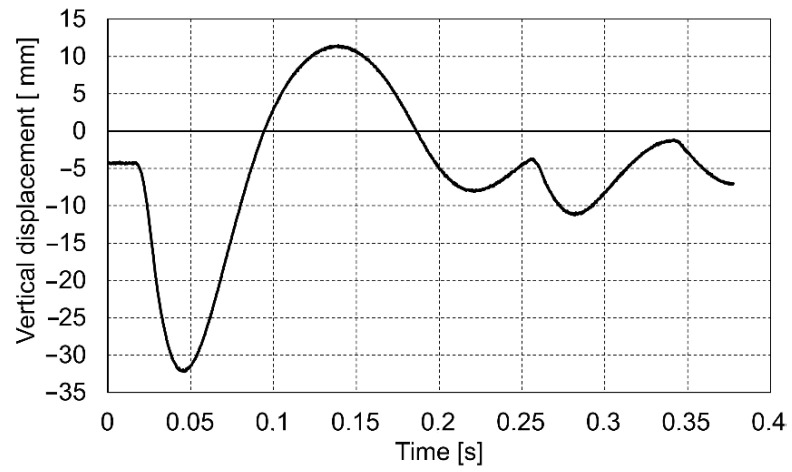
Vertical displacement of the p0 point during drop No. 2.

**Figure 11:**
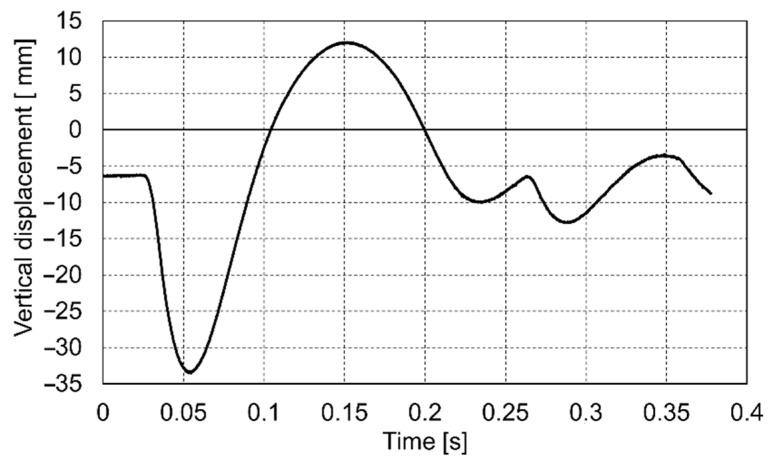
Vertical displacement of the p0 point during drop No. 3.

The course of the impact was recorded in the dynamic plate test with the use of a high-speed camera. On this basis, the values of the settlement of the loaded soil were determined for the entire impact phase. Below are presented the diagrams (Figure 9, Figure 10 and Figure 11) of displacement during three consecutive drops for the p0 point. Positive displacement values on the graphs are related to the detachment of the plate from the tested soil.

The values of the measured displacements at individual points during impacts together with the calculated values of the dynamic modulus of deformation *E_vd_* are shown in Table 4. The rising average values of *E_vd_* from 775.29 kPa (Drop 1) to 832.51 kPa (Drop 3) indicate a slight compaction of the test ground after each drop.

### 4.7. Minimum Values of the Weak Soil Parameters for Test Shooting

Taking into account the similar (dynamic) action of the mortar plate and the dynamic load plate, the dynamic modulus of deformation can be adopted as a $E_{vd}$ parameter characterizing the weak soil in terms of test shooting. Using Equation (1), it is possible to estimate the minimum dynamic modulus of deformation of the weak soil, at which it will be possible to safely fire a mortar. For this purpose, it was assumed the Poisson’s ratio of peat ν=0.2, the diameter of the mortar base plate $d_{m}=33\;\mathrm{cm}$, and ω=0.79 for a circular rigid plate [31]. In addition, a possible displacement of the mortar’s base plate should be assumed, which will allow a safe shot to be fired $\Delta s_{max}=50\;\mathrm{mm}$. The maximum pressure under the base plate of a 60 mm mortar is $\Delta p_{max}=1.57\;\mathrm{MPa}$. Thus, we get:

(2) $$E_{vd}^{min}=\left(1-0.2^{2}\right)\cdot 0.79\cdot \frac{0.33\cdot 1.57\cdot 10^{6}}{0.050}=7.86\;\mathrm{MPa}$$

The weak ground should therefore be prepared in such a way that the dynamic modulus is larger than $E_{vd}^{min}$. Due to the fact that the dynamic plate is not adapted to study the deformation modulus of the weak substrate, when determining the modulus *E_vd_* it is necessary to use a high-speed camera. However, this procedure is difficult to carry out in the field. A simpler test is to evaluate the static modulus on the basis of a static load test. In the case of the proposed type of weak soil, the relationship between the static and dynamic modulus can be determined. The dependency will apply only to the chosen type of soil. In the presented case, the average primary static modulus is E=144.6 kPa (see Table 2). The average dynamic modulus after the third drop is $E_{vd}=832.5\;\mathrm{kPa}$ (see Table 3). Thus, we get a coefficient of proportionality:

(3) $$a=\frac{E_{vd}}{E}=5.8$$

In the case of the tested soil, it can be assumed that the minimum static modulus that must be obtained in order for the substrate to meet the assumed conditions is:

(4) $$E^{min}=\frac{E_{vd}^{min}}{a}=\frac{7.86\;\mathrm{MPa}}{5.8}=1.36\;\mathrm{MPa}$$

At this stage, the coefficient a should be determined each time at the initial stage of selection of the soil because it could be different for various types of organic soil.

In the calculation of the static modulus, the coefficient for a rigid circular plate is used (see Equation (1)). However, it is possible to carry out static test loads using a rigid square-shaped plate. In this case, the shape factor ω in Equation (1) shall be 0.88 [31].

The determined values of the modules for the model soil are lower than the minimum calculated values, however, in the opinion of the authors, verification of the suitability of the proposed soil under the field conditions is required.

## 5. Medium Soil

**Figure 12:**
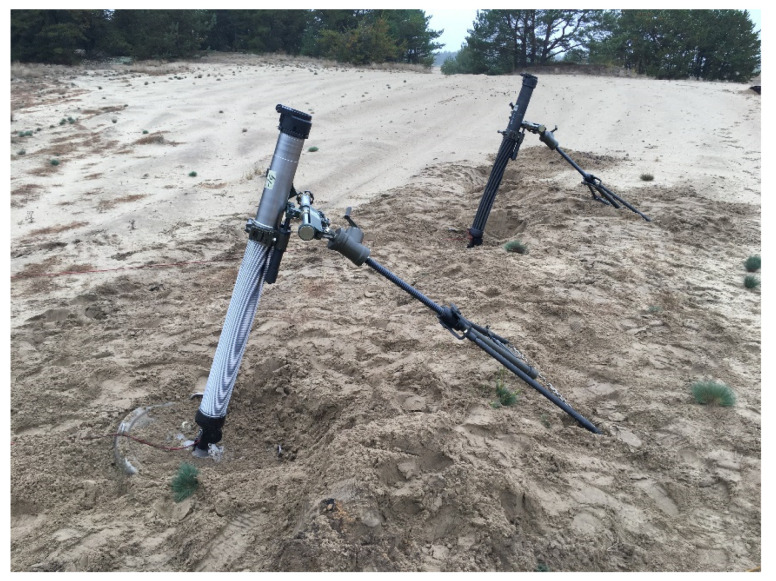
The mortars on the firing position.

To characterize the substrate by the dynamic deformation modulus, preliminary field tests combined with shot tests with a 98 mm mortar were carried out in the field (Figure 12).

According to the standard [19], the medium soils are sandy, clayey, and turf soils. As part of the assessment of the value of the dynamic sandy soil deformation modulus (fine sands—FSa), three tests were conducted with a lightweight deflectometer. The research was carried out within the shooting position at a depth of 0.5 m, after removing the top layers of sandy soil and humus. The determined average values of soil settlement are in the range of 0.527–0.399 mm, and the dynamic modulus in the range of 42.69–56.39 MPa with an average value of 48.17 MPa.

**Figure 13:**
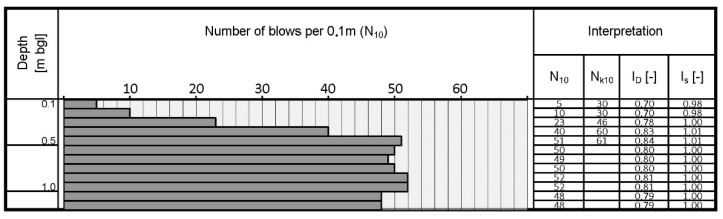
Dynamic probe profile.

As part of the assessment of the density of sandy soil, dynamic probing using DPL was performed. The results of the test are shown in Figure 13.

The value of the density index *I_D_* and the degree of compaction *I_S_* are determined respectively from the relationships:

(5) $$I_{D}=0.429logN_{\left(k\right)10}+0.071\;\;\;\;\;\left[-\right]$$

(6) $$I_{s}=\frac{0.818}{0.958-0.174I_{D}}\;\;\;\;\left[-\right]$$

**Figure 14:**
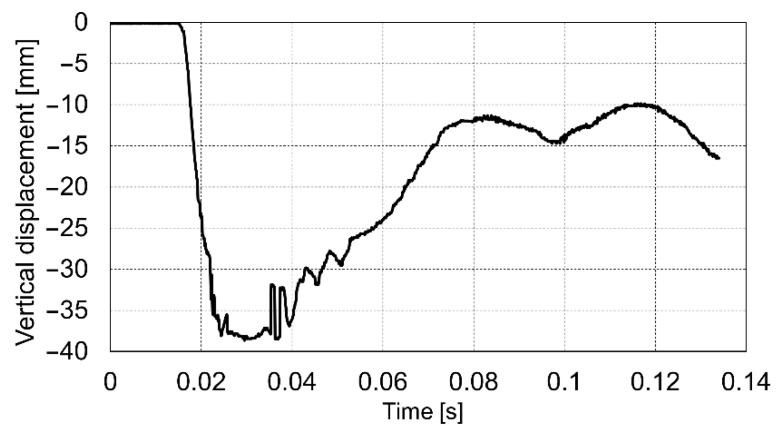
Displacements of the base plate during the third shot.

At the shooting place, four shots were fired. The first two shots were intended to pre-adjust the plate to the ground. The recoil pulses were 2153.84 and 2828.7 (Ns), respectively. The next two shots were carried out on a maximum pressure propelling charge (for mortar’s endurance tests) and the recoil pulse achieved 3704.07 (Ns). The maximum measured displacements of the base plate during shots No. 3 and 4 were 38.44 and 38.67 mm, respectively. The displacement of the base plate during the third shot is shown in Figure 14.

The possible displacement of the base plate which will allow a safe shot to be fired for this type of mortar is equal to 100 mm (19). The measured maximum displacement of the mortar’s base plate is close to the middle of the safe range (about 40% of 100 mm). Thus, the soil with the parameters determined above can be assumed as a medium subgrade for mortar testing.

Using the results of the maximum displacements of the base plate and assuming in Equation (2): ν=0.3 for sand, $d_{m}=80\;\mathrm{cm},$ the value of the dynamic deformation modulus *E_vd_* from the firing pulse load can be determined. The maximum pressure under the base plate of the mortar caliber 98 mm is $\Delta p_{max}=1.9\;\mathrm{MPa}$, and the average maximum displacement from two mortar shots $\Delta s_{śrmax}=38.55\;\mathrm{mm}$, so:

(7) $$E_{vd}=\left(1-0.3^{2}\right)\cdot 0.79\cdot \frac{0.8\cdot 1.9\cdot 10^{6}}{0.03855}=28.3\;\mathrm{MPa}$$

The obtained value of 28.3 MPa is lower than the value obtained from the dynamic plate test (48.17 MPa). The above can be influenced by a number of factors, of which the most important seem to be:

- The tube of the mortar and the base plate during the shot are not perpendicular to each other, which may cause slight lateral displacements of the plate;
- Dependence of the deformation modulus on the stress value;
- The shape factor ω was adopted for a rigid circular plate, which can be a certain approximation in the case of a mortar base plate, which is not perfectly rigid and has special ribbing;
- The soil inertia has higher influence on the speed of deformations during mortar firing than at research tests.

## 6. Conclusions

Designing standardized test stands with mortar test sites is a complex issue. In order to ensure the repeatability of the test conditions, the characteristics of the shooting process and the interaction between the mortar and the soil should be taken into account. It is also important in order to confirm that the mortar meets the requirements, including the safety of its operation. The use of standardized soils (with a parametric description of their geotechnical properties) and practical measurement methods will allow for an unambiguous and correct implementation of the mortar certification process. Due to the specificity of shooting tests, the development of stations and research methods that can be used under the field conditions would be beneficial. At the moment, there are no such stands or research methods.

The research and results presented in the paper constitute the first approach to the development of standardized test stands (including soft soils). The requirements for the stands require an innovative approach to the issue of the object’s impact on the ground in terms of geotechnical engineering. This is caused by both a much wider range of soil properties (including weak soil, not used in construction) and many times higher loads generated by the mortar. In the next stage, experimental verification of methods and calculations will be carried out by conducting a shooting test.

In order to characterize the soil conditions using geotechnical parameters and more accurate methods, the soil moduli of deformation were chosen.

In the case of designing the mortar test stand for weak soils, peat was used as a subgrade material. This kind of soil was chosen because it is widely available and allows for easy preparation of weak soil conditions. However, the stiffness of organic soil is not the subject of geotechnical testing as it is assumed that such soil has very little bearing capacity and should be improved. In order to parametrize the weak soil for mortar testing, the authors proposed the dynamic load test in conjunction with the static load test. The static load test was introduced because the dynamic load test generated large settlements of the plate which had to be determined using a high-speed camera, making the procedure problematic. Thus, the idea was to carry out a much simpler test and determine the static modulus of the peat. Then, the dynamic modulus can be calculated using a previously established empirical Equation between static and dynamic moduli for the used weak soil.

In the case of medium soils, the sandy subsoil was parameterized in the field. The deformation modulus from the lightweight dynamic plate test was determined, as well as the compaction parameters based on DPL probing. In the opinion of the authors, the abovementioned parameters can be used in order to standardize the medium subgrade conditions for mortar testing.

The value of the dynamic modulus of deformation was also calculated based on the displacement of the mortar base plate as a result of the shot impulse. The determined values of this modulus and the dynamic load plate modulus do not coincide, which is due to a number of factors related to the peculiarities of the mortar shot. However, it should be emphasized that the main goal was to parameterize the soil conditions for weak and medium subgrades in relation to mortar testing procedure. Further studies are required to determine the influence of the various factors on the results obtained and to determine a sufficiently accurate correlation between the results obtained by means of the presented research methods and shooting tests.

## 7. Discussion

The carried out tests and their development will allow for more accurate modeling of the process of cooperation of the mortar with the substrate during the shot. This is also essential when designing mortars, including ensuring the correct strength of the base plate, ensuring better stability and accuracy of the mortar, and reducing its weight.

The mortar has to be tested for three different conditions of subgrade: weak, medium, and hard soil. These conditions correspond to the movement of the mortar base plate after shot. When designing a test stand for the weak soil conditions, the movement of the plate after shot must be close to the limiting value designed for the particular mortar type, ensuring safety of its operation. If the settlement of the mortar plate exceeds the safe limit, the mortar might be damaged. Therefore, the soil has to be prepared in such a way that the mortar plate settlement is close to the limit value but does not exceed it. The static or dynamic load modulus can be used in order to prepare such weak soil. The dynamic load modulus for the weak soil test prepared for the preliminary study presented in the paper was between 775.29 kPa and 832.51 kPa. However, based on theoretical calculations (see Equation (2)) the dynamic modulus should be at least 7.86 MPa. Test shootings on this soil will be carried out to verify the assumptions. If the value of the modulus is too low, the peat can be compacted in layers and/or sand can be mixed with peat to decrease its compressibility.

For the medium soil, the goal was to obtain the displacement of the mortar base plate after shot close to the middle of the safe range. For the tested 98 mm mortar, the required range is 0–100 mm. After shooting, the maximum average displacement of the base plate was 38.55 mm. Thus, the soil with the average dynamic modulus close to 48.17 MPa can be assumed as medium subgrade for mortar testing. Additionally, the dynamic modulus based on mortar plate settlement was calculated. It must be admitted that the difference between the value of 28.3 MPa (obtained from mortar shots) and the value of 48.17 MPa (obtained from the dynamic plate test) is significant, which is probably influenced by the factors mentioned previously in the article. In the current study for sandy ground (“medium soil”) the dynamic plate modulus is equal to 1.7 times the mortar’s modulus. The obtained dependence requires confirmation in subsequent planned tests.

Shooting tests using a 60 mm mortar on the proposed weak soil are planned. Additionally, geotechnical parameters of the hard soil will be determined, and shooting tests will be carried out.

An important aspect of the research is the influence of stress level and dynamics of deformations on the soils properties. It should be emphasized that this kind of research has not been reported in the available literature so far.

## Figures and Tables

**Table 1:** The recoil parameters in mortars operated by the Polish Armed Forces (obtained during verification tests of mortars conducted in the Military Institute of Armament Technology).

L. p.	Caliber of Mortar	Recoil Force Pulse (Ns)	Average Duration of the Shot (ms)	Weight of Rejected Elements (kg)	Theoretical Recoil Velocity (m/s)
1	60 mm	320	8	18.3	≈17.5
2	98 mm	3620	10	120	≈30.2
3	120 mm	4400	14	207	≈21.3

**Table 2:** Static load test results.

Phase of Test	Load (kg)	Force (kN)	Pressure (kPa)	Average Value of Settlement (cm)
Loading	16.40 *	0.16	2.28	0.00
	49.80	0.49	6.91	−0.73
	73.80	0.72	10.24	−1.25
	97.40	0.96	13.51	−1.83
	124.90	1.22	17.33	−2.35
	156.40	1.53	21.70	−3.10
	186.50	1.83	25.87	−3.63
Unloading	214.90	2.11	29.81	−4.33
	186.50	1.83	25.87	−4.30
	156.40	1.53	21.70	−4.10
	124.90	1.22	17.33	−3.85
	97.40	0.96	13.51	−3.53
	73.80	0.72	10.24	−3.23
	49.80	0.49	6.91	−2.88
	16.40	0.16	2.28	−1.90

* weight of rigid plate.

**Table 3:** Primary static deformation modules.

Pressure *p* (kPa)	Increase in Pressure Δ*p* (kPa)	Settlement *s* (cm)	Increase in Settlement Δ*s* (cm)	Primary Static Deformation Modulus E_1_ (kPa)	
2.28	0.00	−0.00	0.00	0.00	144.6 (average)
6.91	4.63	−0.73	0.73	144.3	
10.24	3.33	−1.25	0.52	145.7	
13.51	3.27	−1.83	0.58	128.3	
17.33	3.82	−2.35	0.52	167.1	
21.70	4.37	−3.10	0.75	132.6	
25.87	4.17	−3.63	0.53	179.0	
29.81	3.94	−4.33	0.7	128.1	

**Table 4:** The values of measured displacements and dynamic modulus of deformation.

	Point p0	Point p1	Point p2
**Drop 1**			
Displacement [mm]	29.48	29.21	29.35
Dynamic modulus of deformation *E_vd_* [kPa]	771.78	778.91	775.19
	775.29 (average)		
**Drop 2**			
Displacement [mm]	27.94	28.14	28.30
Dynamic modulus of deformation *E_vd_* [kPa]	814.31	808.52	803.96
	808.93 (average)		
**Drop 3**			
Displacement [mm]	27.14	27.43	27.42
Dynamic modulus of deformation *E_vd_* [kPa]	838.32	829.46	829.76
	832.51 (average)		

## Data Availability

Data sharing is not applicable to this article.
